# Curing Kinetic Parameters of Epoxy Composite Reinforced with Mallow Fibers

**DOI:** 10.3390/ma12233939

**Published:** 2019-11-28

**Authors:** Lucio Fabio Cassiano Nascimento, Fernanda Santos da Luz, Ulisses Oliveira Costa, Fábio de Oliveira Braga, Édio Pereira Lima Júnior, Sergio Neves Monteiro

**Affiliations:** 1 Military Institute of Engineering, Rio de Janeiro 22290-270, Brazil; lucio_coppe@yahoo.com.br (L.F.C.N.); ulissesolie@gmail.com (U.O.C.); edio@ime.eb.br (É.P.L.J.); snevesmonteiro@gmail.com (S.N.M.); 2 Fluminense Federal University, Rio de Janeiro 24210-240, Brazil; fabio_obraga@yahoo.com.br

**Keywords:** polymer matrix composites, thermosetting resin, curing behavior, thermal analysis

## Abstract

Knowledge about the curing behavior of a thermosetting resin and its composites includes the determination of kinetic parameters and constitutes an important scientific and technological tool for industrial process optimization. In the present work, the differential scanning calorimetry (DSC) technique was used to determine several curing parameters for pure epoxy and its composite reinforced with 20 vol % mallow fibers. Analyses were performed with heating rates of 5, 7.5, and 10 °C/min, as per the ASTM E698 standard. The kinetic related parameters, that is, activation energy (E), Avrami’s pre-exponential factor (Z), and mean time to reach 50% cure (t½), were obtained for the materials, at temperatures ranging from 25 to 100 °C. Response surfaces based on the mathematical relationship between reaction time, transformed fraction, and temperature were provided for optimization purposes. The results showed that the average curing time used for the production of diglycidyl ether of bisphenol A/triethylenetetramine (DGEBA/TETA) epoxy systems or their composites reinforced with natural mallow fibers can be considerably reduced as the temperature is increased up to a certain limit.

## 1. Introduction

**Figure 1:**
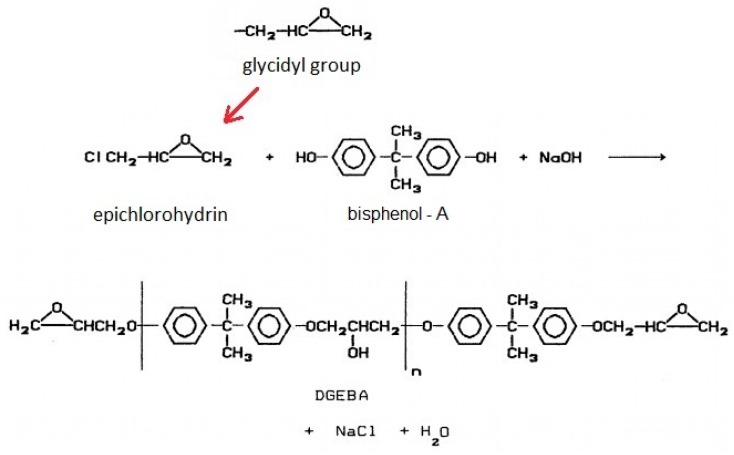
Production reaction of the epoxy resin [2] (adapted).

Epoxy resins are thermosetting polymers characterized by the presence of glycidyl groups in their macromolecules. By reacting the glycidyl group with a suitable crosslinking agent (hardener), a three-dimensional structure is formed. Epichlorohydrin (1-chloro-2,3-epoxy-propane) is the universal agent bearing the epoxy group that reacts with chemical species that have active hydrogens. Bisphenol A is the most common chemical species that possesses these active hydrogens [1]. Figure 1 illustrates the reaction to produce the most common type of epoxy resins, the diglycidyl ether of bisphenol A, also known as DGEBA [2].

Epoxy resins can be used for several applications. High-performance adhesives are some of the most popular. These are polymers applied to join other polymers, metals, ceramics, or composites. In this particular case, the resin is classified as chemically reactive adhesive, in which the epoxy is composed of a two-component system, going through the curing process when the components are mixed [1,2].

Some epoxy characteristics that are worth mentioning include excellent combination of mechanical properties, good electrical properties, dimensional stability, effective adherence, excellent corrosion resistance, and low cost. Typical applications are as adhesives (as already mentioned), electrical molds, components for the automotive and aerospace sectors, protective coatings, sports articles, as well as a polymer matrix for composites with fiber reinforcement [2].

Investigation on the kinetics of the curing reaction of plain epoxy resin or as the polymer matrix in composites has been conducted by different areas [3,4,5,6,7,8,9]. Razak and Emad [6] reported the curing kinetics of the diglycidyl ether of bisphenol A/triethylenetetramine (DGEBA/TETA) system with different hardener/resin ratios (phr), 5, 13, and 20, using the differential scanning calorimetry (DSC) technique. They measured isothermal curing at four temperatures (30, 45, 60, and 80 °C) and found that the complete cure occurs at 80 °C at 13 phr, a ratio that is used in the present work, which is the stoichiometric ratio. Unlike the above work [6], the purpose of this one was to find the time when complete cure occurs with the incorporation of natural mallow fiber, in order to optimize the time for composite production, without compromising their complete cure. The rheological properties of the isothermal curing of epoxy resin have been reported [7]. In another recent work, Ferdosian et al. [8] investigated the incorporation of lignin-based epoxy resins derived from depolymerized Kraft/organosolv lignins into a conventional DGEBA resin at different fractions to prepare bio-based epoxy systems as polymer matrices for the manufacturing of fiber-reinforced composites. They found that the epoxy composites with 25 wt % of lignin-based epoxy resin cured faster than the pure DGEBA resin. This may be explained by the viscosity increase with higher amounts of lignin-based epoxy resin, which impairs the hydroxyl groups’ effect, hence increasing the activation energies and slowing down the curing process [8]. The slowing down of the curing process in an epoxy resin was also observed by Abenojar et al. [9] when nano-carbon particles were added into the epoxy matrix.

Currently, with the growing demand for sustainable materials, natural fiber-reinforced composites are being considered to replace traditional synthetic materials, such as fiberglass, in several applications [10,11,12,13,14,15,16,17,18,19,20,21,22,23,24]. Among the natural reinforcing fibers, mallow fibers obtained from a plant of the malvaceous species (*Urena lobata* Linn.) stand out due to their high strength-to-weight ratio and very low cost. These fibers are produced in local communities in the Brazilian part of the Amazon forest [22].

In the production of epoxy matrix composites reinforced with natural fibers [10,11,12,13,14], after mixing the polymer and the fibers in the desired ratio, the conventional curing time used is 24 hours [15,16,17,18,19,20,21,22,23,24]. However, this time may be significantly different from that required for the complete curing reaction of the polymer matrix and therefore of the composite to be manufactured.

In the present work, the differential scanning calorimetry (DSC) analysis was performed to evaluate if the total curing time of the epoxy resin could be less than the previously reported time of 24 h [15,16,17,18,19,20,21,22,23,24]. This would optimize the availability of equipment such as presses and metal molds, as well as reduce the total production costs. In addition, with respect to natural fiber-reinforced composites, it is of scientific interest to understand how these natural fibers modify the curing kinetic parameters of the epoxy.

Thus, the objective of the present work was to obtain the curing kinetic parameters for an epoxy DGEBA resin, in order to understand, for the first time, the influence of the addition of natural mallow fibers (20 vol %) on the curing behavior of the epoxy DGEBA/TETA system.

## 2. Materials and Methods 

Commercial epoxy resin diglycidyl ether of bisphenol A (DGEBA) was investigated in the present work. It was hardened for the experiments using the stoichiometric ratio of triethylenetetramine (TETA). Both substances were acquired from the Brazilian company Epoxyfiber Chemical Industry (Rio de Janeiro, Brazil).

**Figure 2:**
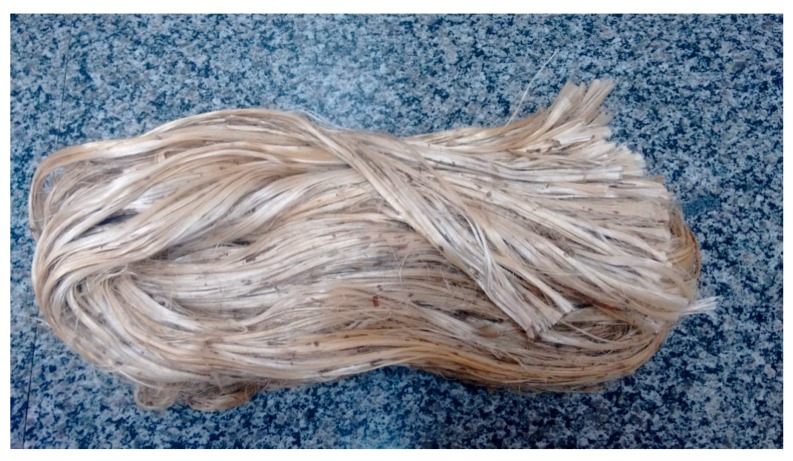
As-received mallow fibers.

The mallow fibers were generously provided by researchers from the State University of North Fluminense (UENF), with the indication that the fibers were acquired from the Brazilian Textile Company Castanhal (Belém, Brazil). Figure 2 illustrates the as-received mallow fibers.

For the differential scanning calorimetry (DSC) analysis, the resin and hardener were mixed with 13 parts of hardener for each 100 parts of resin (phr = 13), and the mixture was placed in an aluminum crucible. Mallow fibers were added to prepare composite specimens with a 20 vol % fraction, to investigate the fiber influence on the epoxy curing kinetics. The reason for selecting that fraction to produce the composite samples was due to the adhesion between the matrix and fiber, which could be impaired with higher volume fractions of reinforcement. This best value has obtained during preliminary testing discussed elsewhere [15]. In that work [15], a very effective attachment was revealed between the broken fibers and the epoxy matrix for the composite with a 20 vol % fraction of mallow fiber. To prepare the mallow fiber/epoxy composites, the mallow fibers were first milled resulting in short fibers, less than ≈ 1 mm long, and then incorporated into the epoxy matrix in randomly dispersed form. Plain epoxy specimens were also investigated as control. The DSC equipment used was a model 404 F1 Pegasus Netzsch, belonging to the Military Institute of Engineering (IME). The analysis was performed in a nitrogen atmosphere with heating rates of 5, 7.5, and 10 °C/min, at a temperature range of 20 to 400 °C. The method described by the ASTM E698 standard was used to calculate the following kinetic curing parameters: activation energy (*E*, kJ/mol), Avrami’s pre-exponential factor (*Z*, min^−1^), rate constant for each temperature (k, min^−1^), and the mean cure time (t_½_, min) for the pure epoxy and the mallow fiber-reinforced composite, at temperatures of 25 to 100 °C. Using the times of 30, 60, 120, 180, 240, and 300 min, and a transformed fraction interval ranging from 50% to 95%, the cure temperatures were found for each specific situation. Based on the mathematical relationships between reaction time, transformed fraction, and temperature, curves with respect to the pure epoxy and the epoxy composite reinforced with 20 vol % of mallow fibers were plotted.

## 3. Results

**Figure 3:**
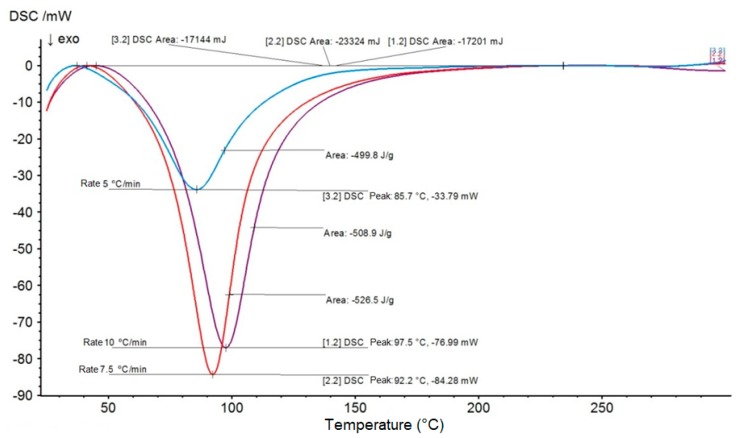
Differential scanning calorimetry (DSC) curves for plain epoxy resin at the heating rates of 5, 7.5, and 10 °C/min, for determination of curing kinetic parameters.

**Figure 4:**
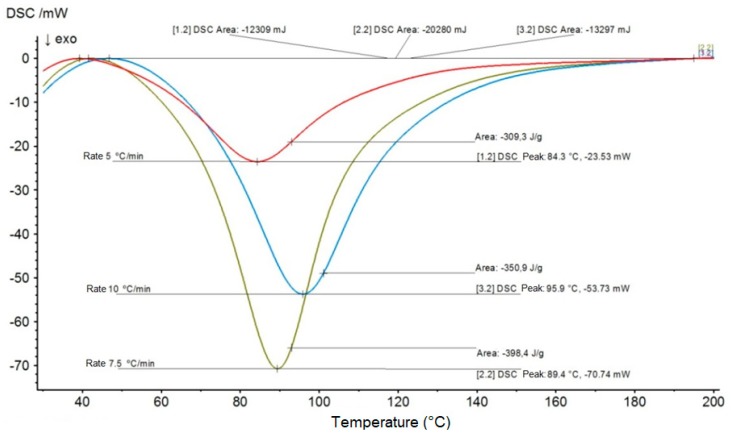
DSC curves for the epoxy composite reinforced with 20 vol % of mallow fibers at the heating rates of 5, 7.5, and 10 °C/min, for determination of curing kinetic parameters.

Figure 3 and Figure 4 show the DSC graphs of temperature (°C) vs heat flow (mW) at the rates of 5, 7.5, and 10 °C/min, which were used to determine the curing kinetic parameters for the aforementioned materials, namely, plain epoxy and 20 vol % mallow fiber composite.

Table 1 shows the results obtained for the activation energy (*E*, kJ/mol), pre-exponential factor (*Z*, min^−1^) and mean time to reach 50% cure (t_½_, min) for plain epoxy and its composite reinforced with mallow fibers, at temperatures ranging from 25 to 100 °C.

The results in Table 1 show in all cases that, as the temperature increases, the mean time t_½_ is reduced. This result was already expected, since the cure of thermosetting resins is a thermally activated process and thus can be modeled by the Arrhenius equation [25]. Despite that fact, for a particular temperature, the specimen with mallow fibers showed a relatively larger reduction in t_½_. This fact can be justified by the fibers acting as nucleating sites on the curing process [4].

According to the ASTM E698 standard and others [4,5,26], the curing process of a polymer resin is modeled as a homogeneous cooling event in which the exponent of the Avrami’s equation “n” was considered as a unit [27]. Equation (1) shows the Avrami’s complete relationship:

(1) $$1-X_{tranf.}=e^{-kt^{n}},$$

where *X_transf_*_._ is the transformed fraction; *k* is the constant rate for each temperature (min^−1^); t is the transformation time (cure) in minutes; and n is Avrami’s exponent (*n* = 1 for ASTM E698).

In order to adjust a mathematical relationship combining time, transformed fraction, and temperature, Equation (2), which correlates the constant rate (*k*) and the pre-exponential factor (*Z*), the following equation is used:

(2) $$k=Ze^{-\left(\frac{E}{RT}\right)},$$

where k is the constant rate for each temperature (min^−1^); *Z* is the pre-exponential factor (min^−1^); *T* is the cure temperature in Kelvin; *E* is the activation energy (J/mol); and *R* = 8.314 J/mol.K.

By combining Equations (1) and (2), the final equation for reaction time, transformed fraction, and temperature is obtained:

(3) $$T=\frac{-E}{Rln\left(\frac{ln\left(1-X_{transf.}\right)}{-Zt}\right)}.$$

Table 2 presents the results obtained from Equation (3) considering the intervals of time (30, 60, 120, 180, 240, and 300 min) and a transformed fraction (50%, 60%, 70%, 80%, 90%, and 95%) as well as the temperature range from 25 to 100 °C for plain epoxy. The final temperatures were transformed from Kelvin to Celsius for convenience.

**Figure 5:**
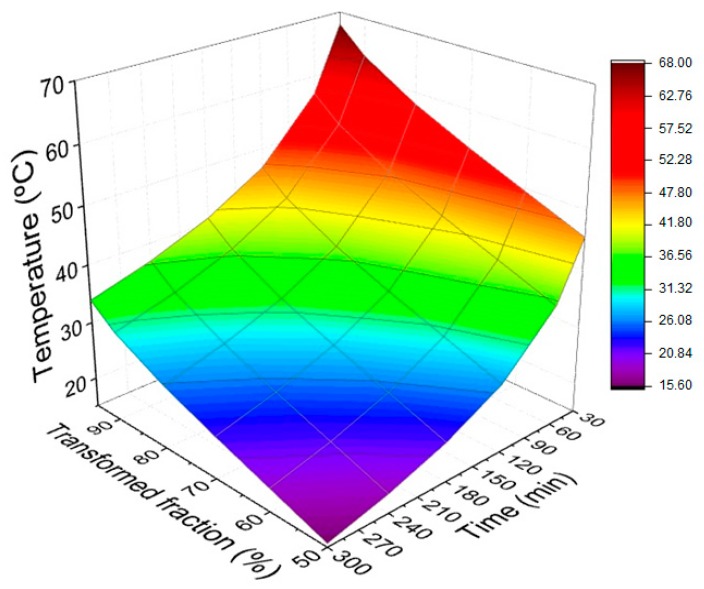
Time curve, transformed fraction, and correlated temperatures (plain epoxy resin).

Figure 5 is a response surface obtained from the results in Table 2. This figure shows the relationship between time, transformed fraction, and temperature, which can be used in practical applications involving the curing reaction of the plain epoxy resin. To find out how much time must be used to perform the curing process at a particular temperature, one can choose the desired transformed fraction for a particular application.

Table 3 was also obtained by means of Equation (3) for the epoxy composite reinforced with 20 vol % of mallow fibers. It can be observed that, as compared to Table 2, for a particular combination of time and transformed fraction, one needs a lower curing temperature. This is attributed to the mallow fibers acting as nucleation sites, accelerating the reactions and favoring the progress of the curing process. To the knowledge of this work’s authors, this fact has not yet been reported in the scientific literature. As before, the final temperatures were transformed from Kelvin to Celsius.

**Figure 6:**
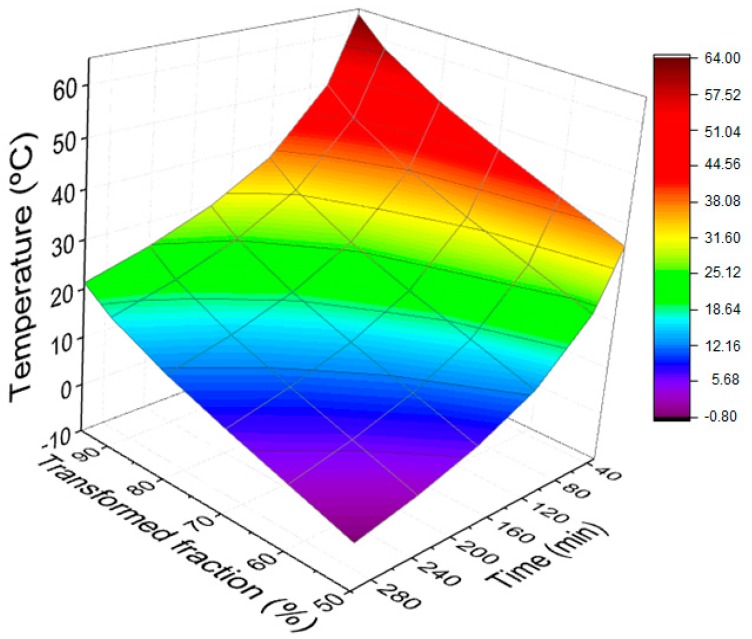
Time curve, transformed fraction, and correlated temperatures (epoxy composite reinforced with 20 vol % of mallow fibers).

Similar to Figure 5, Figure 6 is a response surface showing the relationship between time, transformed fraction, and temperature for the epoxy composite reinforced with 20 vol % of mallow fibers.

The most striking difference between the response surface in Figure 5 and Figure 6 is that the curing temperatures for the epoxy composite reinforced with 20 vol % of mallow fibers are sensibly lower than those for the plain epoxy. To the knowledge of this work’s authors, this fact is reported for the first time in the literature.

## 4. Conclusions

In the present work, the curing kinetics of a diglycidyl ether of bisphenol A/triethylenetetramine (DGEBA/TETA) epoxy system and of its composite reinforced with 20 vol % of mallow fibers was studied, following procedures indicated by the ASTM E698 standard.

- Kinetic parameters, such as the activation energies (58.971 kJ/mol and 44.536 kJ/mol), the pre-exponential factors *Z* (1.08 × 10^8^ min^−1^ and 8.04 × 10^5^ min^−1^), and the times to reach 50% of the curing reaction (t_½_), for temperatures ranging from 25 to 100 °C were calculated.
- Particularly for the commonly used room temperature (25 °C), the values of t_½_ 138.68 and 55.26 min for curing were found for epoxy resin and the composite reinforced with 20 vol % mallow fibers, respectively.
- The curves’ response surfaces obtained for both plain epoxy and 20 vol % mallow fiber-reinforced composites have practical application in the determination of the appropriate combination of time, transformed fraction, and temperature, according to the actual case.
- Curing temperatures for the composite reinforced with 20 vol % mallow fibers are lower than that for pure epoxy resin, for a particular time. This possibly means that natural fibers act as catalysts for the curing reaction, a finding which is reported for the first time for a natural fiber-reinforced epoxy composite.

## Figures and Tables

**Table 1:** Composite reinforced with 20 vol % of mallow fibers.

Test Materials	Activation Energy (kJ/mol)	Pre-exponential Factor “*Z*” (min^−1^) × 10^5^	Temperatures (°C)	Constant Rate “k” (min^−1^)	Mean Time to Reach 50% Cure “t_½_” (min)
Plain epoxy resin	58.97	1.080	25	0.0050	138.68
			50	0.0316	21.98
			80	0.2039	3.40
			100	0.5987	1.16
Epoxy composite reinforced with 20 vol % of mallow fibers	44.54	8.036	25	0.0125	55.26
			50	0.0504	13.75
			80	0.2064	3.36
			100	0.4656	1.49

**Table 2:** Time, transformed fraction, and correlated temperatures (plain epoxy resin).

Transformed Fraction (%)	Time (min)					
	30	60	120	180	240	300
50	45.49 °C	35.87 °C	26.82 °C	21.77 °C	18.29 °C	15.64 °C
60	49.53 °C	39.67 °C	30.40 °C	25.23 °C	21.67 °C	18.96 °C
70	53.58 °C	43.48 °C	33.99 °C	28.69 °C	25.05 °C	22.28 °C
80	58.01 °C	47.64 °C	37.89 °C	32.47 °C	28.73 °C	25.89 °C
90	53.58 °C	43.48 °C	33.99 °C	28.69 °C	25.05 °C	22.28 °C
95	67.89 °C	56.90 °C	46.60 °C	40.86 °C	36.92 °C	33.93 °C

**Table 3:** Time, transformed fraction, and correlated temperatures (epoxy composite reinforced with 20 vol % of mallow fibers).

Transformed Fraction (%)	Time (min)					
	30	60	120	180	240	300
50	35.49 °C	23.65 °C	12.68 °C	6.63 °C	2.50 °C	−0.63 °C
60	40.53 °C	28.30 °C	17.00 °C	10.77 °C	6.51 °C	3.29 °C
70	45.62 °C	33.00 °C	21.35 °C	14.93 °C	10.55 °C	7.24 °C
80	51.22 °C	38.16 °C	26.12 °C	19.50 °C	14.97 °C	11.56 °C
90	58.40 °C	44.77 °C	32.22 °C	25.33 °C	20.63 °C	17.08 °C
95	63.88 °C	49.81 °C	36.87 °C	29.77 °C	24.92 °C	21.27 °C

## References

[1] Brydson J.A. (1999). Plastics Materials.

[2] Askeland D.R., Phulé P.P. (2006). The Science and Engineering of Materials.

[3] Montserrat S., Málek J. (1993). A kinetic analysis of the curing reaction of an epoxy resin. Thermochim. Acta.

[4] Costa M.L., Rezende M.C., Pardini L.C. (1999). Methods of study of curing kinetics of epoxy resins (in Portuguese, Brazil). Polímeros.

[5] Menezes G.W., Monteiro S.N., D’almeida J.R.M., Neto H.S.N. (2004). Thermal analysis of DGEBA / TETA epoxy resin for different formulations of stoichiometric ratio (in Portuguese). ABM.

[6] Razak A.A.A., Emad M. (2016). Cure Kinetics of Epoxy Resin Studied by Dynamics and Isothermal DSC Data. Eng. Technol. J. A.

[7] Razak A.A.A., Emad M. (2015). The Rheological Properties of Isothermal Curing of DGEBA/TETA Epoxy System. Eng. Technol. J. A.

[8] Ferdosian F., Zhang Y., Yuan Z., Anderson M., Xu C.C. (2016). Curing kinetics and mechanical properties of bio-based epoxy composites comprising lignin-based epoxy resins. Eur. Polym. J..

[9] Abenojar J., del Real J.C., Ballesteros Y., Martinez M.A. (2018). Kinetics of curing process in carbon/epoxy nano-composites. IOP Conf. Ser. Mater. Sci. Eng..

[10] Mohanty A.K., Misra M., Hinrichsen G. (2000). Biofibres, Biodegradable Polymers and Bio-composites: An overview. Macromol. Mater. Eng..

[11] Sahed D.N., Jog J.P. (1999). Natural fiber polymer composites: A review. Adv. Polym. Technol..

[12] Bledzki A.K., Gassan J. (1999). Composites reinforced with cellulose based fibers. Prog. Polym. Sci..

[13] Monteiro S.N., Lopes F.P.D., Barbosa A.P., Bevitori A.B., Da Silva I.L.A., Da Costa L.L. (2011). Natural lignocellulosic fibers as engineering materials—An overview. Metall. Mater. Trans. A.

[14] Moraes Y.M., Ribeiro C.G.D., Ferreira C.L., Lima E.S., Margem J.I., Nascimento L.F.C., Monteiro S.N. (2018). Mechanical behavior of mallow fabric reinforced polyester matrix composites. J. Mater. Res. Technol..

[15] Nascimento L.F.C., Monteiro S.N., Louro L.H.L., Luz F.S., Santos J.L., Braga F.O., Marçal R.L.S.B. (2018). Charpy impact test of epoxy composites reinforced with untreated and mercerized mallow fibers. J. Mater. Res. Technol..

[16] Nascimento L.F.C., Holanda L.I.F., Louro L.H.L., Monteiro S.N., Gomes A.V., Júnior E.P.L. (2017). Natural Mallow Fiber-Reinforced Epoxy Composite for Ballistic Armor Against Class III-A Ammunition. Metall. Mater. Trans. A.

[17] Pereira A.C., Monteiro S.N., Assis F.S., Margem F.M., Luz F.S., Braga F.O. (2017). Charpy impact tenacity of epoxy matrix composites reinforced with aligned jute fibers. J. Mater. Res. Technol..

[18] Nascimento L.F.C., Louro L.H.L., Monteiro S.N., Junior E.P.L., Luz F.S. (2017). Mallow Fiber-Reinforced Epoxy Composites in Multilayered Armor for Personal Ballistic Protection. JOM.

[19] Maciel N.O.R., Ferreira J.B., Vieira J.S., Ribeiro C.G.D., Lopes F.P.D., Margem F.M., Monteiro S.N., Vieira C.M.F., Silva L.C. (2018). Comparative tensile strength analysis between epoxy composites reinforced with curaua fiber and glass fiber. J. Mater. Res. Technol..

[20] Braga F.O., Milanezi T.L., Monteiro S.N., Louro L.H.L., Gomes A.V., Lima J.R.E.P. (2018). Ballistic comparison between epoxy-ramie and epoxy-aramid composites in Multilayered Armor Systems. J. Mater. Res. Technol..

[21] Nascimento L.F.C., Louro L.H.L., Monteiro S.N., Gomes A.V., Marçal R.L.S.B., Lima J.R.E.P., Margem J.I. (2017). Ballistic Performance of Mallow and Jute Natural Fabrics Reinforced Epoxy Composites in Multilayered Armor. Mater. Res.-Ibero-Am. J..

[22] Margem J.I., Gomes V.A., Margem F.M., Ribeiro C.G.D., Braga F.O., Monteiro S.N. (2015). Flexural Behavior of Epoxy Matrix Composites Reinforced with Malva Fiber. Mater. Res.-Ibero-Am. J..

[23] Monteiro S.N., Assis F.S., Ferreira C.L., Simonassi N.T., Weber R.P., Souza M.O., Colorado H., Pereira A.C. (2018). Fique Fabric: A Promising Reinforcement for Polymer Composites. Polymers.

[24] Luz F.S., Tommasini F.J., Nascimento L.F.C., Figueiredo A.B.S., Monteiro S.N. (2018). Critical length and interfacial strength of PALF and coir fiber incorporated in epoxy resin matrix. J. Mater. Res. Technol..

[25] Callister W.D., Rethwish D.G. (2012). Materials Science and Engineering—An Introduction.

[26] D’Almeida J.R.M., Menezes G.W., Monteiro S.N. (2003). Ageing of the DGEBA/TETA epoxy system with off-stoichiometric compositions. Mater. Res.-Ibero-Am. J..

[27] Avrami M. (1939). Kinetics of Phase Change. I General Theory. J. Chem. Phys..

